# The Impact of Acute Tinnitus on Listening Effort: A Study Based on Clinical Observations of Sudden Sensorineural Hearing Loss Patients

**DOI:** 10.3390/ijerph19063661

**Published:** 2022-03-19

**Authors:** Chii-Yuan Huang, Dian-Sian Li, Ming-Hsien Tsai, Chih-Hao Chen, Yen-Fu Cheng

**Affiliations:** 1 Department of Otolaryngology Head and Neck Surgery, Taipei Veterans General Hospital, Taipei 112, Taiwan; dopod0635@gmail.com (C.-Y.H.); lightboyc2007@gmail.com (D.-S.L.); michaelchen808@gmail.com (C.-H.C.); 2 Faculty of Medicine, National Yang-Ming Chiao-Tung University, Taipei 112, Taiwan; 3 Department of Speech Language Pathology and Audiology, National Taipei University of Nursing and Health Sciences, Taipei 112, Taiwan; ksmworm@gmail.com; 4 Department of Medical Research, Taipei Veterans General Hospital, Taipei 112, Taiwan; 5 Institute of Brain Science, National Yang Ming Chiao Tung University, Taipei 112, Taiwan

**Keywords:** listening effort, tinnitus, sudden sensorineural hearing loss

## Abstract

This study investigates the relationship between listening effort and acute tinnitus over the clinical course of sudden sensorineural hearing loss (SSNHL) before and after treatment. Thirty SSNHL patients with acute tinnitus were enrolled in this prospective study. Each patient was evaluated before treatment and after 1 and 3 months of follow-up. Listening effort was evaluated in the unaffected ears in two conditions (with and without background noise) using a dual-task paradigm, which included a primary (speech recognition) task and a secondary (visual reaction time) task. Tinnitus severity was assessed with the Tinnitus Handicap Inventory (THI). It was observed that background noise significantly increased listening effort in SSNHL patients with acute tinnitus before and after treatment. THI scores and listening effort in quiet conditions (** *p* = 0.009) were significantly decreased three months after treatment. In an analysis of the relation between tinnitus severity and listening effort, it was found that the THI total score was significantly correlated with listening effort in quiet (* *p* = 0.0388) and noisy conditions (* *p* = 0.044) before treatment. We concluded that SSNHL patients with acute tinnitus exerted greater listening effort in the presence of background noise than in quiet conditions. Furthermore, listening effort was reduced as tinnitus improved in SSNHL patients during the three months after treatment. Both before and after 3 months of treatment, patients who were more affected and emotionally distressed by tinnitus tended to exert more listening effort in both quiet and noisy environments.

## 1. Introduction

Listening effort is defined as the cognitive resources used to understand speech [1]. In other words, it describes the degree of effort that listeners need to expend to comprehend a speaker’s wording. Listening effort increases with the number of challenges hindering listeners from understanding what they are hearing. Several factors or acoustic challenges, such as hearing impairment, background noise, and unfamiliar language or accent, affect listening efforts [2]. Clinically, patients with sensorineural hearing loss often complain of tinnitus, which also causes emotional distress and impairs cognitive function [3,4,5]. Under such circumstances, tinnitus patients might need to allocate additional effort to speech recognition, i.e., increase their listening effort [6].

Although the presence of tinnitus has been found to be associated with hearing loss, some tinnitus cases are accompanied by normal hearing sensitivity [7,8], which implies the possibility of subclinical cochlear impairment [9]. Tinnitus is thought to be a form of internal noise experienced by listeners; however, its relationship with listening effort is still unclear [2,10]. The literature contains contradictory reports concerning the effect of tinnitus on listening effort. One study by Degeest et al. demonstrated that chronic tinnitus increased listening effort in young adults with normal hearing [10]. In contrast, Jensen et al. reported that tinnitus participants showed reduced pupil dilation while listening, indicating a reduction in the amount of effort needed for speech recognition [11].

Idiopathic sudden sensorineural hearing loss (SSNHL) is defined as hearing loss of at least 30 dB at 3 sequential frequencies within 3 days with no identifiable causes [12]. Despite extensive research efforts, controversy still remains in the etiology of idiopathic SSNHL. Some frequent causes have been suggested, including infection, trauma, noise/blast exposure, stroke, tumor/malignancy, and ototoxic agents [12,13,14]. SSNHL is an otological emergency and a distressing disease that may cause disability [15,16]. Other clinical symptoms include tinnitus and vertigo [17,18]. In addition to hearing impairment, approximately one-third of patients with SSNHL suffer from severe tinnitus or hyperacusis [18,19,20]. The prevalence of tinnitus in SSNHL patients is approximately 79% to 97% [21,22], and its severity may change as patients’ hearing status fluctuates during treatment [23]. The improvement of hearing is a favorable prognostic factor for the recovery from tinnitus-related disability in SSNHL [24].

The effect of tinnitus on listening effort remains unclear, but the existing clinical observations suggest that the change in tinnitus status with hearing recovery in SSNHL patients could provide an interesting model to evaluate the effect of acute tinnitus on listening effort. Because tinnitus is a form of internal noise that may affect speech recognition in both ears through central interference, measurements can be made specifically on the unaffected side to eliminate the direct influence of hearing loss on the lesioned side [25]. We hypothesized that listening effort would change with tinnitus in these patients. Thus, the purpose of this study was to investigate the impact of acute tinnitus on listening effort in SSNHL before and after the course of treatment.

## 2. Materials and Methods

### 2.1. Study Participants

This prospective study was conducted between April 2018 and June 2019, and patients diagnosed with SSNHL at a tertiary referral hospital were enrolled in this study. All the recruited patients had sudden hearing loss in one ear with concomitant tinnitus, and the unaffected ear had normal hearing (−10 to 19.9 dB HL) or mild hearing impairment (20 to 34.9 dB HL) according to the Global Burden of Disease Hearing Loss Expert Group’s definition of hearing impairment [26]. Patients with head and/or neck trauma or barotrauma, chronic tinnitus history, inflammatory ear diseases, retrocochlear lesions, or neurocognitive disorders and exposure to ototoxic drugs were all excluded from the study. Routine serological, auditory, and electro-neurophysiological tests would be arranged during the admission period to exclude non-idiopathic causes [12]. All patients received treatment in our hospital. The standard protocol for SSNHL included intravenous dexamethasone (5 mg/mL) twice per day for 5 days, followed by oral methylprednisolone tapered every 2 days for 6 days. Demographic data, including age, sex, and affected side, were collected. This study was approved by the Institutional Review Board of Taipei Veterans General Hospital (2017-11-006BC).

### 2.2. Audiological Tests

Acoustic immittance of tympanogram was performed to exclude possible middle ear pathology for all the patients. Vestibular examinations, including the electronystagmography, were arranged for patients with vertigo complaints. Audiological tests, including pure-tone audiometry (PTA) and speech recognition threshold (SRT), were measured at the pretreatment period (T0) and 1 month (T1) and 3 months (T3) after treatment. The mean pure-tone thresholds for air conduction (AC) were presented as AC1 by averaging 500, 1000, 2000, and 4000 Hz and as AC2 by averaging 500, 1000, 2000, 4000 and 8000 Hz. During the treatment period, an auditory brain stem response test was also conducted for each patient to exclude retrocochlear lesions. The degree of hearing improvement after treatment was assessed by Siegel’s criteria [27], as follows: final hearing better than 25 dB HL was classified as type I (complete recovery); final hearing of 25–45 dB HL with >15 dB improvement was classified as type II (partial recovery); final hearing poorer than 45 dB HL but with >15 dB improvement was classified as type III (slight improvement); <15 dB improvement was classified as type IV (no improvement).

### 2.3. Tinnitus Measurements

The severity of tinnitus was evaluated at time points T0, T1, and T3 with a Mandarin version of the Tinnitus Handicap Inventory (THI) [28]; the original THI was introduced by Newman et al. in 1996 [29]. In this study, patients were matched for the loudness based on a visual analog scale from 1 to 10. The THI is a self-administered questionnaire that consists of 25 items, and each item is grouped into functional, emotional, and catastrophic subscales. Patients are asked to complete each item with three scoring answers: no (0 points), sometimes (2 points), and yes (4 points). The THI total score (the sum of 25 item scores) ranges from 0 to 100, and scores are categorized as follows: little or no handicap (0–16 points) is grade 1, a mild handicap (18–36 points) is grade 2, a moderate handicap (38–56 points) is grade 3, a severe handicap (58–76 points) is grade 4, and a catastrophic handicap (78–100 points) is grade 5.

### 2.4. Dual-Task Paradigm

**Figure 1:**
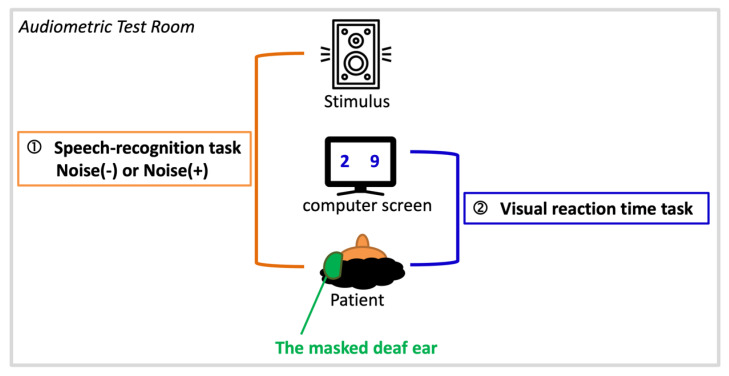
Schematic illustration of a dual-task paradigm composed of a primary task-speech recognition task in quiet (noise−) or noise (noise+) and a secondary task-visual reaction time task.

Listening effort was assessed by a dual-task paradigm (Figure 1) as modified by Picou et al. [30]. This paradigm is a proven and well-established procedure, and there are many reports in the literature verifying its feasibility and reliability as a measure of cognitive load, with the auditory (primary) task and visual (secondary) task competing for limited cognitive resources [31,32]. Our paradigm consists of a primary task and a secondary task, both of which were carried out in an audiometry testing room. To eliminate the impact of the degree of hearing loss in the affected ears on listening effort, all patients’ affected ears were masked before tasks to ensure that they were in a single-side hearing condition with similar hearing levels (i.e., under normal or mild hearing impairment of the unaffected ear).

The dual-task paradigm started with a primary task, which was a speech recognition task. The speech stimulus was presented through a loudspeaker 1 meter in front of the patients. The stimuli were phonetically balanced words spoken by male narrators in Mandarin [33]. The sound pressure levels of the stimuli were controlled by a GSI 61 clinical audiometer (Grason Stadler Inc., Eden Prairie, MN, USA) and were set to 40 dB above the speech recognition threshold of the unaffected ear to ensure adequate sound stimulation.

Patients were presented with randomly ordered recordings of 48 spondaic Chinese words in spoken form and then asked to repeat what they had heard. There were two listening conditions: quiet and background noise (signal-to-noise ratio = 0 dB). The accuracy of patients’ responses was monitored and recorded by the researcher.

A secondary task was presented immediately (125 ms) after the primary task. This task was a visual reaction time task, in which two numbers from 1 to 9 appeared on computer screens placed in front of patients at a distance of 0.45 meters, and patients were required to judge which number was greater than the other by pressing a keyboard button as quickly as possible. This dual-task program was designed with E-Prime 2.0 software (Psychology Software Tools, Sharpsburg, PA, USA) in the Windows XP operating system.

Before each test at T0, T1, and T3, each subject was assigned a pretest consisting of 12 dual tasks to ensure that the individual was familiar with the demanding procedure, in which a primary (auditory) speech recognition test is followed shortly (125 ms) by a visual selection task. In our preliminary test experience, most subjects failed in the first 3–5 dual tasks because of an unfamiliarity with the procedure or because of inattention. Their responses became more stable after 10–12 dual tasks (the pretest), i.e., the patients were able to perform at their best; then, we began to test for listening effort through the primary task (speech recognition of Chinese spondee words) and secondary task. The presentation order of the words was randomly selected by our program from a preset list of 48 spondee words. Each secondary task result (reaction time) was monitored by our audiologist to check whether the primary task (speech recognition) was correctly answered. If the subject failed in the primary task (i.e., repeated the spondee words incorrectly), the secondary task result was not included in the analysis. The listening effort results (reaction time) were determined when at least 20 dual tasks had been correctly completed, resulting in at least 20 data points from which to calculate the average reaction time. Our program also included a labeling function to mark any inattention or fatigue, indicated by success in the primary task followed by an excessively delayed response in the secondary task; a response was defined as excessively delayed if its latency was over 2 standard deviations longer than the mean latency. These data points were labeled by the program, and the researchers considered them outliers in terms of reaction time.

### 2.5. Statistical Analysis

Paired-sample t-tests or ANOVAs were used to evaluate the pre- and post-treatment audiological data, THI scores, and dual-task reaction time. The Spearman rank correlation test was used to evaluate the association between THI scores and reaction time as well as the association between tinnitus loudness and reaction time. The intraclass correlation coefficient was used to estimate the test-retest reliability of the dual-task paradigm.

## 3. Results

### 3.1. Patients’ Characteristics and Audiological Results

Thirty patients were included in the study. The age of the patients was 46.6 ± 16.3 years (mean ± standard deviation, SD; range: 16–65). Of the patients, 18 (60%) were male and 12 (30%) were female. The affected ear was on the left side in 10 (33%) patients and on the right side in 20 (67%) patients. Unilateral tinnitus was noted in all patients in their affected ears. The duration between the onset of symptoms to treatment ranges from 2 days to 2 months (60 days), with an average of 8.5 days (SD ± 12.0), as shown in Table 1.

For the unaffected ears, the mean PTA thresholds of AC1 at T0, T1, and T3 were 18.53 ± 9.58 dB HL, 19.02 ± 11.33 dB HL, and 21.18 ± 10.24 dB HL, respectively, with no significant difference between the three time points. The mean PTA thresholds of AC2 at T0, T1, and T3 were 25.40 ± 21.17 dB HL, 21.71 ± 13.28 dB HL, and 14.13 ± 11.94 dB HL, respectively, with no significant difference among the three time points. The mean SRTs at T0, T1, and T3 were 15.71 ± 9.41 dB HL, 16.04 ± 11.01 dB HL, and 16.39 ± 6.14 dB HL, respectively (Table 2).

For the lesioned ears, the mean pretreatment (T0) PTA threshold of AC1/AC2 was 53.13 dB HL (SD ± 26.13)/62.07 dB HL (SD ± 20.97), whereas one month after the treatment (T1), it was 39.64 dB HL (SD ± 21.27)/42.71 dB HL (SD ± 20.95), with significant improvement (AC1: * *p*< 0.05; AC2: ** *p* < 0.01) (Table 1). The mean pretreatment SRT was 59.52 dB HL (SD ± 25.57), which improved significantly to 34.02 dB HL (SD ± 23.21) at T1 (** *p* < 0.01). Six patients were lost to follow-up three months after the treatment (T3), while the remaining (n = 24) patients’ mean PTA threshold of AC1/AC2 and mean SRT were 38.76 dB HL (SD ± 20.50)/41.83 dB HL (SD ± 21.28) and 27.92 dB HL (SD ± 20.56), respectively. Compared with the mean PTA threshold at T0, the mean PTA threshold of AC2 and the mean SRT improved significantly at T3; the mean PTA threshold of AC1 showed a trend of improvement, but the trend was not significant (*p* = 0.058). The PTA threshold of each frequency at T0, T1, and T3 was analyzed using ANOVA for the lesioned and unaffected ears to show the significant changes during the treatment course (Table 3). Regarding hearing improvement at T3, the remaining 24 patients included 6 patients showing complete recovery, 3 showing partial recovery, 3 showing slight improvement, and 12 showing no improvement according to Siegel’s criteria.

### 3.2. Severity of Tinnitus—THI Scores and Loudness Matching

**Figure 2:**
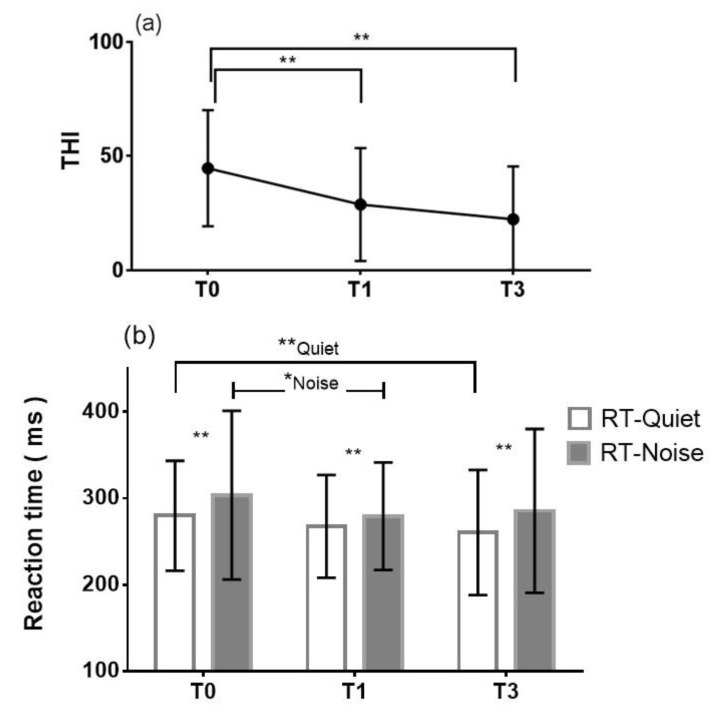
(**a**) Mean THI total score and (**b**) mean reaction time in quiet and with noise at pretreatment (T0), 1 month after treatment (T1) and 3 months after treatment (T3). THI = Tinnitus Handicap Inventory; RT-Quiet = mean reaction time in quiet conditions; RT-Noise = mean reaction time with noise. * *p* < 0.05. ** *p* < 0.01.

The THI was used to evaluate the severity of tinnitus at the pretreatment period (T0) and 1 month (T1) and 3 months (T3) after treatment, as shown in Figure 2a. The mean pretreatment THI total score was 44.7 ± 25.4 (mean ± SD) (as moderate tinnitus), with a range from 10 to 88, and it improved significantly (** *p* < 0.0001) to 28.7 ± 24.8 (as mild tinnitus) at T1. The mean THI total score at T3 was 22.3 ± 23.2 (as mild tinnitus), with a range from 0 to 64, which also showed significant improvement (** *p* < 0.0001) compared with the score at T0.

**Figure 3:**
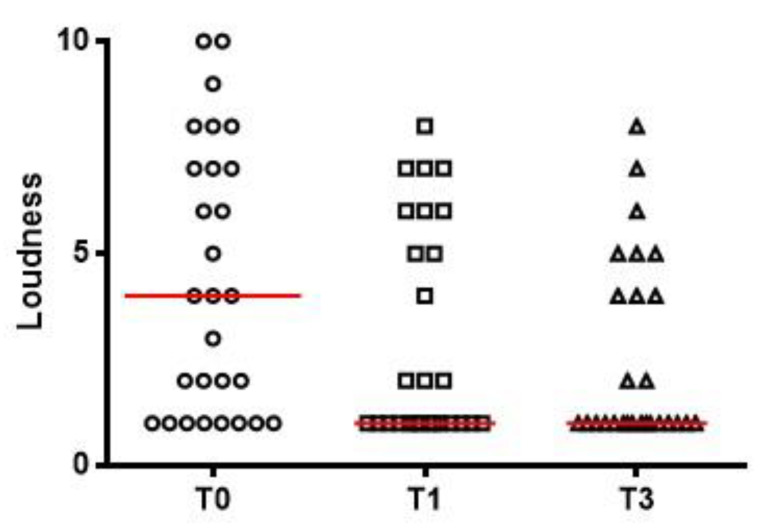
Tinnitus loudness as measured by a visual analog scale before treatment (T0, range 1 to 10, median 4), 1 month after treatment (T1, range 1 to 7, median 1) and 3 months after treatment (T3, range 1 to 7, median 1). Red lines represent median values.

**Figure 4:**
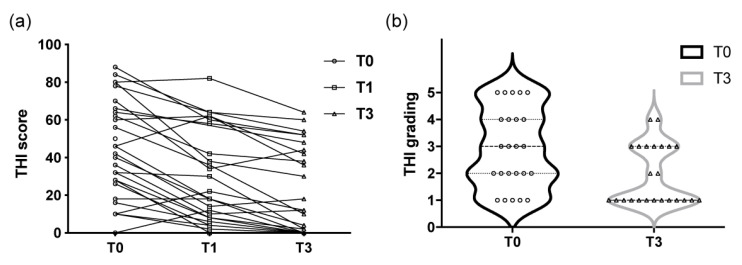
(**a**) The trends in each patient’s THI score over time. (**b**) THI grading before and after treatment; (T0) before treatment, (T1) 1 month after treatment, and (T3) 3 months after treatment. THI = Tinnitus Handicap Inventory.

Tinnitus loudness values are shown in Figure 3, including visual analog scale scores at T0 (ranging from 1 to 10 with a median of 4), T1 (1 to 7, median: 1) and T3 (1 to 7, median: 1). The changes in each patient’s THI score and THI grading over the course of the study are shown in Figure 4.

### 3.3. Listening Effort of the Unaffected Ear—Dual-Task Performance

Listening effort of the unaffected ear was assessed by a dual-task paradigm at different time points (T0, T1 and T3), as shown in Figure 2b. The mean pretreatment reaction time in quiet conditions was 279.8 ± 63.5 ms and decreased to 267.5 ± 59.4 ms (mean ± SD) at T1, showing no significant difference (*p* = 0.16). However, with background noise, the mean pretreatment reaction time was 303.5 ± 97.5 ms, which decreased significantly (* *p* = 0.036) to 279.3 ± 62.1 ms at T1. At T3, only the mean reaction times in quiet conditions were significantly shorter than those at T0 (** *p* = 0.009); with background noise, there was no significant difference (*p* = 0.11). At all three time points (T0, T1, and T3), the mean reaction time was significantly longer with background noise than in quiet conditions, and the difference was statistically significant (T0: ** *p* = 0.0029; T1: ** *p* = 0.0023; T3: ** *p* = 0.0054). The intraclass correlation coefficient for evaluating the reliability of the dual task was 0.83, indicating good test-retest reliability.

### 3.4. Correlations between Tinnitus and Dual-Task Performance

The relation between THI and listening effort (dual-task performance) was investigated using Spearman correlation coefficients (Table 4). At T0, the THI total score was significantly correlated with dual-task performance either in quiet conditions (r = 0.3791, * *p* = 0.0388) or with noise (r = 0.3906, * *p* = 0.0440), while the correlations at T1 and T3 showed no significance. These significant correlations were also evident in the emotional subscales at T0 and T3, both in quiet conditions (T0: r = 0.5040, ** *p* = 0.0045; T3: r = 0.5232, ** *p* = 0.0179) and with noise (T0: r = 0.4333, * *p* = 0.024; T3: r=0.5462, ** *p* = 0.0127). The relation between tinnitus loudness (visual analog scale) and listening effort is also shown in Table 4. These significant correlations were found only at T3, both in quiet conditions (r = 0.4915, * *p* = 0.0278) and with noise (r = 0.5144, * *p* = 0.0203). These individual correlations are also shown in scatter plots in Appendix A and Appendix B, including the correlations of reaction time with THI total scores, all 3 subscales (C, E, F), and loudness. The Spearman correlation coefficient (r) values and p values are indicated in each graph for reference.

## 4. Discussion

This is the first study to investigate the relationship between listening effort and acute tinnitus by observing the clinical course in SSNHL cases. Patients with SSNHL and acute tinnitus exhibited greater listening effort under background noise conditions than under quiet conditions; listening effort was reduced as tinnitus improved in SSNHL patients three months after treatment. We also found that before treatment, patients’ THI scores were significantly correlated with their listening effort both in quiet conditions and with noise, especially in the emotional subscales.

### 4.1. Measurement of Listening Effort

To eliminate the direct influence of hearing loss at the lesion sites, listening effort was evaluated unilaterally in the unaffected ears in our study. Listening effort is the amount of cognitive resources needed to understand an acoustic speech signal. There are many methods for measuring listening effort, including subjective questionnaires, behavior measures such as dual tasks, physiologic measures (cortisol level and pupillometry), and neuroimaging such as electroencephalogram and functional magnetic resonance imaging (fMRI) [2,34]. The dual-task paradigm is a common and widely accepted procedure and was therefore selected as a tool for measuring listening effort in this study [35]. Dual-task measuring is based on the limited resource capacity theory proposed by Kahneman in 1973 [36]: the total cognitive resources of an individual person are limited in capacity. The dual-task paradigm usually comprises a primary task (auditory) and a secondary task (reaction). In our study, a visual reaction task (judgment of number size) was used as the secondary task. Thus, based on Kahneman’s theory, more resources spent on the primary task would leave fewer remaining resources available for secondary tasks, resulting in poor performance or delayed response on secondary tasks. This delayed response on secondary tasks would result in prolonged reaction time between tasks, indirectly indicating increased listening effort on primary tasks. Unlike previous studies testing binaural hearing, our SSNHL patients in the dual-task paradigm were tested monaurally by masking their affected ears. Different degrees of hearing level in the affected ears of the participants could also interfere with the correlation between tinnitus and listening effort; therefore, we tested only the unaffected ears to decrease the impact of hearing loss from the lesion sides. Through these attention-demanding tasks, our reaction time represented the listening efforts in SSNHL patients under unilateral hearing of their unaffected sides.

### 4.2. Factors Influencing Listening Effort

Listening effort can be affected by a variety of factors. When listeners are hearing impaired or of advanced age, listening effort can be increased, as it is difficult for hearing-impaired listeners to process acoustic signs clearly due to impaired acoustic ability, and it is also challenging for elderly people to remember and recognize speech due to declines in cognitive function [37,38]. To overcome these obstacles of signal processing, listeners have to spend more listening effort clarifying the meanings of each spoken word and sentence. Another common factor increasing listening effort is environmental background noise. One study showed that adults with normal hearing had longer response times on a dual-task paradigm in background noise conditions than in quiet conditions [39]. In our study, background noise also negatively affected the performance of SSNHL on secondary tasks with increasing reaction time in both pre- and post-treatments. For people with good or poor hearing, background noise seems to somehow increase their listening effort. Because the speech signal is degraded by noise, listeners spend more energy meeting the cognitive demands of understanding speech, which consequently results in more listening effort.

### 4.3. Changes in Tinnitus and Listening Effort during the Clinical Course

**Figure A2:**
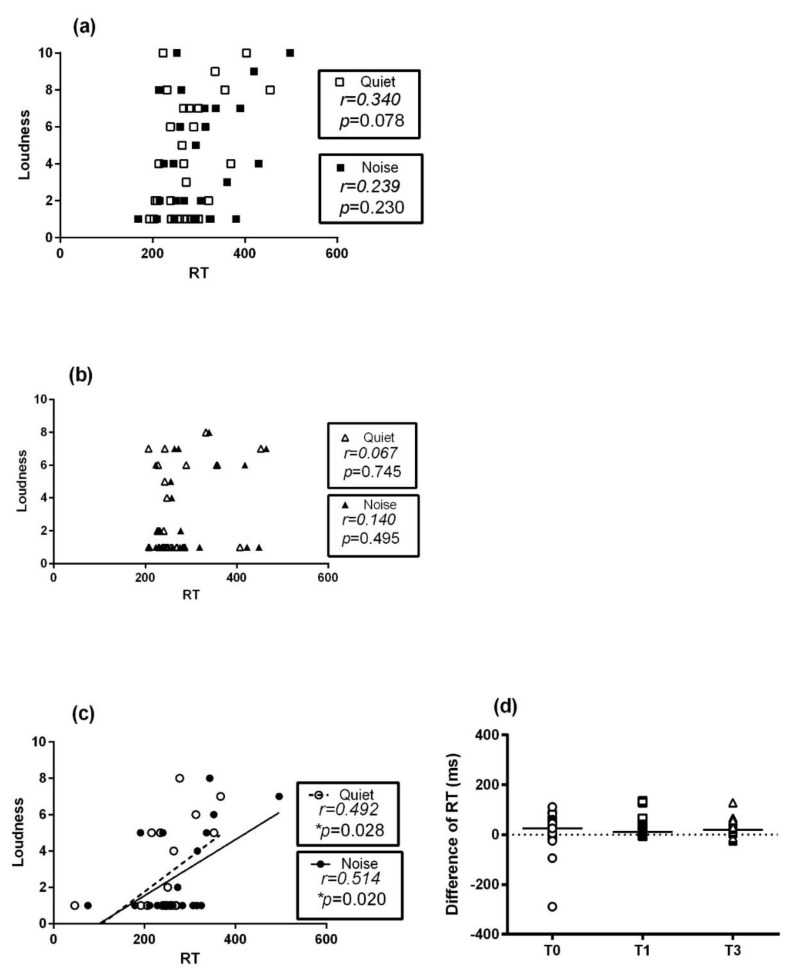
The correlation between tinnitus loudness and reaction time (**a**) before treatment, T0; (**b**) 1 month after treatment, T1; and (**c**) 3 months after treatment, T3. (**d**) Scatter plots of tinnitus loudness and the differences in reaction time between noise and quiet at T0, T1, and T3. RT: reaction time; r: Spearman correlation coefficient; * *p* < 0.05.

Like background noise, tinnitus, which is regarded as internal noise, may distort or interfere with external acoustic signals. To assess whether tinnitus contributes to the masking of speech stimuli in the same manner that background noise does, one can test whether there are larger differences between noise and quiet performance with lower tinnitus (i.e., after treatment) and smaller differences in situation when tinnitus is higher (i.e after treatment). A scatter plot of tinnitus loudness and the differences in reaction time between noisy and quiet conditions is shown in Figure A2d; the correlation is not statistically significant. In addition, some authors have shown that tinnitus may affect working memory and attention, which are crucial cognitive processes for speech recognition [10]. As per our hypothesis, changing tinnitus before and after treatment might affect listening effort as well. Unlike previous studies that focused on “chronic” tinnitus and listening effort at a specific timepoint [10,11], we evaluated how “acute” tinnitus affected listening effort in 3 clinical periods. In our study, tinnitus severity was improved from T0 to T1, but listening effort was not significantly decreased in the quiet condition. However, under the noise condition, the reaction time at T0 was significantly longer than that at T1. According to Figure 2b, the reaction times decrease significantly only in quiet conditions from T0 to T3 and in noisy conditions from T0 to T1. Therefore, the reaction times do not coincidently change with time over the disease course. This could be explained by the fact that combining background noise and tinnitus at early time course (i.e., T0 to T1) tended to cause a great burden for SSNHL patients to complete auditory tasks, demanding more listening effort. At T3, when tinnitus was greatly improved, these patients showed less listening effort than at T0, especially in quiet conditions. Similar results were also found with loudness matching and listening effort at T3, both in quiet conditions and with noise.

### 4.4. Relationship between Acute Tinnitus and Listening Effort

Degeest et al. found that normal hearing participants with tinnitus exhibited more effort than control participants (without tinnitus), but the degree of effort was not correlated with the total THI score in either quiet or noisy conditions [10]. However, our study showed that the THI total scores as well as the emotional subscale scores were significantly correlated with reaction time before treatment, whether in quiet or noisy conditions. Based on the data shown in Table 4, we concluded that listening effort correlates with the THI total score only at T0 and not at T1 or T3. By examining the subscales, we found that the emotional subscores at T0 and T3 also correlated with listening effort. The reason why the correlation was lost at T1 and then returned at T3 could be as follows.

The decreases in tinnitus and listening effort showed an asynchronous trend. Our results showed that most patients had considerable improvements in their tinnitus as well as their listening effort in quiet conditions at T3 (Figure 2a,b). In contrast, from T0 to T1, only listening effort in noisy conditions showed a significant difference. This might be explained as additive effects of tinnitus and noise on listening effort. When tinnitus continued to improve through T3, the asynchronous decrease in listening effort in quiet might reflect only a significant correlation with the emotional subscale and not other subscales or total THI scores. In other words, at T3, the decreases in the functional and catastrophic subscores had a considerable effect on the THI total score, which rendered the correlation with listening effort nonsignificant. However, the emotional subscale remained significantly related to listening effort. Specifically, when patients were more emotionally distressed by tinnitus, they needed more time to complete the dual task. Similar results could also be found at T3, at which time tinnitus loudness was significantly related to listening effort.

This could potentially be explained by our choice to measure loudness with a visual analog scale, which is subjective and susceptible to emotional influence. Clinically, SSNHL patients with continuous tinnitus were more likely to be emotionally distressed than those without tinnitus [40]. In our study, the degree of emotional distress reflected by the THI emotional subscale showed a significantly positive correlation with listening effort.

### 4.5. Limitations of This Study

There are some limitations to our study. First, it would be difficult to recruit a control group of healthy volunteers available for repeated testing in a 3-month duration. However, we have included the (binaural) listening effort of 28 healthy, tinnitus-free adults in our preliminary data (Appendix C). In summary, the listening effort in the preliminary data is slightly smaller than our patients’ results, but not significantly. The differences may be due to the differences in subject age and testing procedure (younger vs. older; binaural vs. monaural). The lack of a control group data did leave some questions to be answered, for example, regarding possible learning effects and coincident changes over time. The effect of learning in this study would be negligible because of the attention-demanding tasks, the pretest arrangement, and the random presentation of primary tasks. Our dual task is a very attention-demanding procedure; for each test (T0, T1, T3) we believe it is best to ensure that each subject is sufficiently familiar with the whole procedure. In other words, the effect of learning over the time course could be minimal or negligible because the subject would enter every trial well prepared and would learn to deal with the demanding tasks. Therefore, the reaction time could be used as a quantified index to present listening effort indirectly. Second, we did not strictly check each patient’s visual acuity, which might have some impact on the performance of the visual reaction time test on the dual-task paradigm. However, we set up a large-screen monitor with large figure presentations to decrease the visual demand of the test. Finally, listening effort might be age-related [37]. Elderly individuals have increased listening effort due to their decreased cognitive function. Therefore, we limited the age of recruited patients to a range of 16 to 65 to avoid the confounding effect of cognitive function. Third, the lack of acufenometry to determine the pitch and intensity of tinnitus is another limitation of this study. Alternatively, we performed a visual analog scale (1 to 10) of tinnitus loudness evaluation. Fourth, the masking noise used in the protocol may have affected the characteristics of the subjects’ tinnitus while performing the dual-task paradigm. In other words, for subjects with mild-to-moderate SSNHL, the effect of masking regarding the characteristics of tinnitus might have been larger than those with severe SSNHL. In this regard, subjects with very similar hearing thresholds on the lesioned ears could have been a better study group.

## 5. Conclusions

Our study is the first to explore the relationship between acute tinnitus and listening effort in SSNHL before and after treatment. Omitting the lesioned ears to reduce the influence of hearing loss, we demonstrated that SSNHL patients with acute tinnitus had greater listening effort in the presence of background noise than in a quiet environment. Furthermore, listening effort was reduced as tinnitus improved after treatment. Both before and after 3 months of treatment, patients who were more prone to being affected and emotionally depressed by tinnitus tended to exhibit greater listening efforts, whether in quiet or noisy conditions.

## Figures and Tables

**Table 1:** The demographic data.

Characteristics	Data
Case (Male:Female)	30 (18:12)
Age (years old)	16–65
mean ± SD	46.6 ± 16.3
Lesion ear (Right:Left)	20:10
Duration (day)	2–60
mean ± SD	8.5 ± 12.0
median	4
Other symptoms	
tinnitus	30
vertigo/dizziness	4/4

**Table 2:** Audiometric results of hearing thresholds (dB HL) of the lesioned and unaffected ears at pretreatment (T0), 1 month after treatment (T1) and 3 months after treatment (T3). AC1 = mean air-conduction threshold at 0.5, 1, 2 and 4 kHz; AC2 = mean air-conduction threshold at 0.5, 1, 2, 4 and 8 kHz; SRT = speech recognition threshold.

		T0	T1	T3
AC1	Lesioned	53.13 ± 26.13	39.64 ± 21.27	38.76 ± 20.50
	Unaffected	18.53 ± 9.58	19.02 ± 11.33	21.18 ± 10.24
AC2	Lesioned	62.07 ± 20.97	42.71 ± 20.95	41.83 ± 21.28
	Unaffected	25.40 ± 21.17	21.71 ± 13.28	14.13 ± 11.94
SRT	Lesioned	59.52 ± 25.57	34.02 ± 23.21	27.92 ± 20.56
	Unaffected	15.17 ± 9.41	16.04 ± 11.01	16.39 ± 6.14

**Table 3:** Analysis (ANOVA) of each frequency for the lesioned and unaffected ears before treatment (T0), 1 month after treatment (T1), and 3 months after treatment (T3). * *p* < 0.05. ** *p* < 0.01. *** *p* < 0.001.

	Hz	250	500	1k	2k	4k	8k
Lesioned side	F	10.79	10.93	10.76	15.34	5.17	8.34
	*p*	0.0014 **	0.0011 **	0.0012 **	0.0002 ***	0.0195 *	0.0035 **
Unaffected side	F	0.2286	0.1482	0.5819	0.4179	0.4136	0.4401
	*p*	0.77	0.86	0.56	0.69	0.69	0.65

**Table 4:** Correlations of THI scores (total and all 3 subscales) and loudness with reaction time in quiet conditions and with noise before treatment (T0), 1 month after treatment (T1) and 3 months after treatment (T3). THI = Tinnitus Handicap Inventory, T: total score; C: catastrophic, E: emotional; F: functional subscales; r: Spearman correlation coefficient; * *p* < 0.05, ** *p* < 0.01.

			r/*p* Value		
			T0	T1	T3
Quiet	THI	T	0.3791/* 0.0388	−0.0058/0.9775	0.3837/0.0949
		C	0.2476/0.1871	0.9165/0.6561	0.2528/0.2822
		E	0.5040/** 0.0045	0.0821/0.6900	0.5232/* 0.0179
		F	0.3112/0.0942	−0.0137/0.9469	0.4058/0.0758
	Loudness		0.3395/0.0772	0.0671/0.7446	0.4915/* 0.0278
Noise	THI	T	0.3906/* 0.0440	0.0049/0.8136	0.4135/0.0700
		C	0.3316/0.0911	0.1182/0.5653	0.2761/0.2387
		E	0.4333/* 0.0240	0.0984/0.6449	0.5462/* 0.0127
		F	0.2629/0.1853	0.0604/0.7694	0.4335/0.0562
	Loudness		0.2389/0.2301	0.1402/0.4945	0.5144/* 0.0203

**Figure A1:**
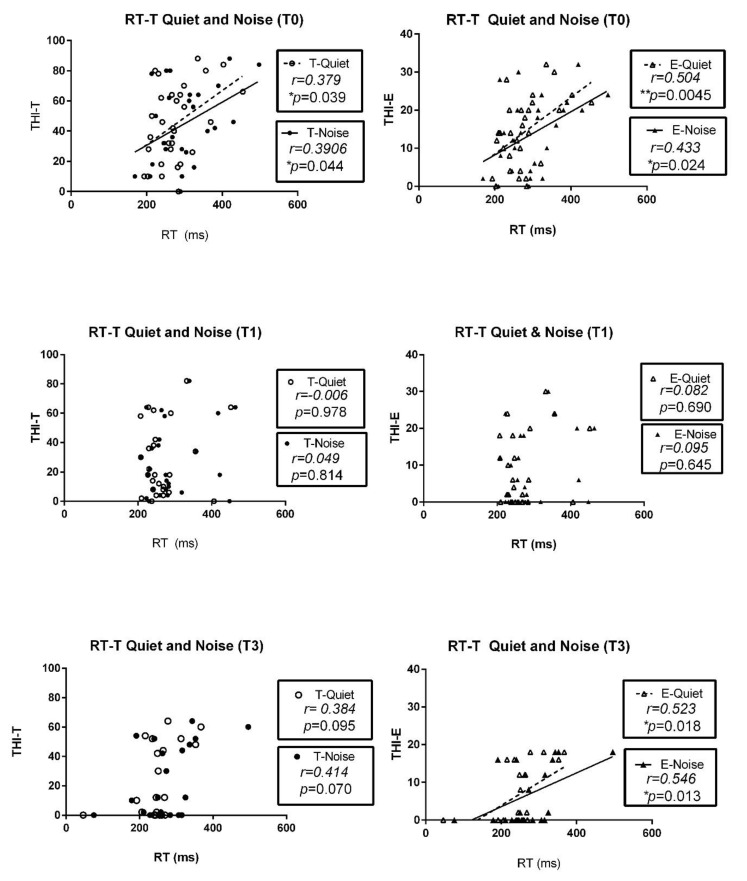

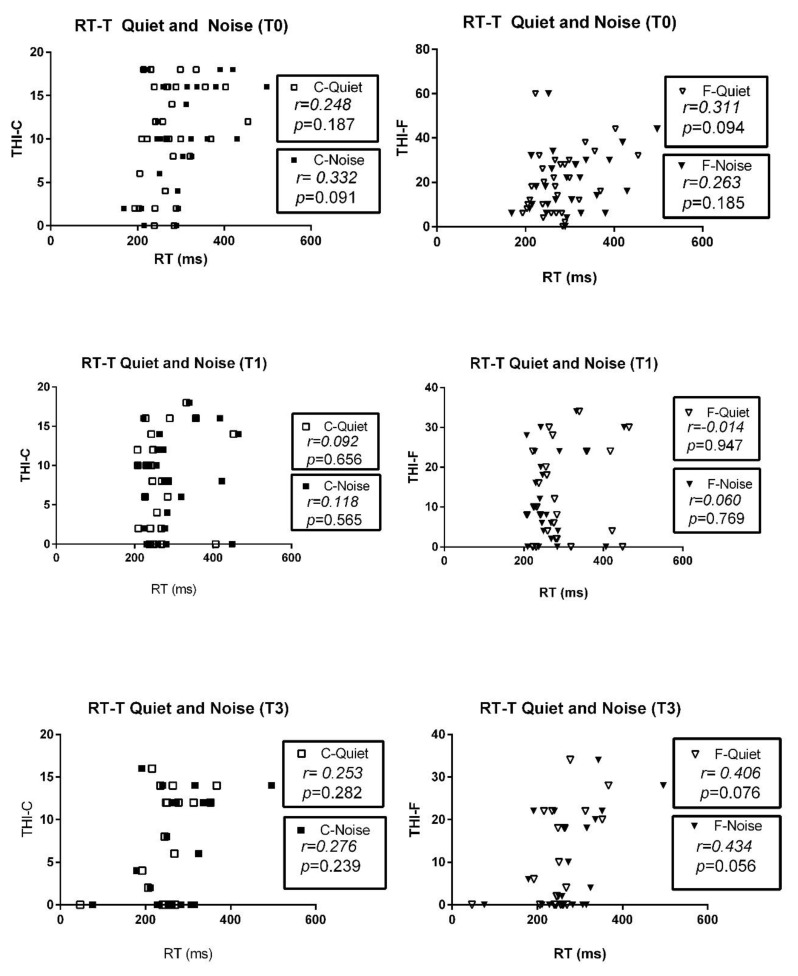
Scatter plots showing the correlation of THI total (T) and subscale scores (C: catastrophic; E: emotional; F: functional) with reaction time (RT); r: Spearman correlation coefficient; * *p* < 0.05, ** *p* < 001.

**Figure A3:**
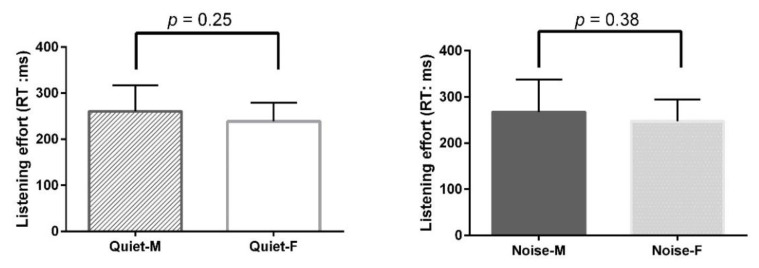
Comparison of Listening effort between male (14) and female (14) subjects. Quiet male: 260.62 ± 55.82 ms, Quiet female: 238.97 ± 39.98 ms, (*p* = 0.25); Noise male: 267.40 ± 69.69 ms, Quiet female: 247.15 ± 47.12 ms, (*p* = 0.38).

**Figure A4:**
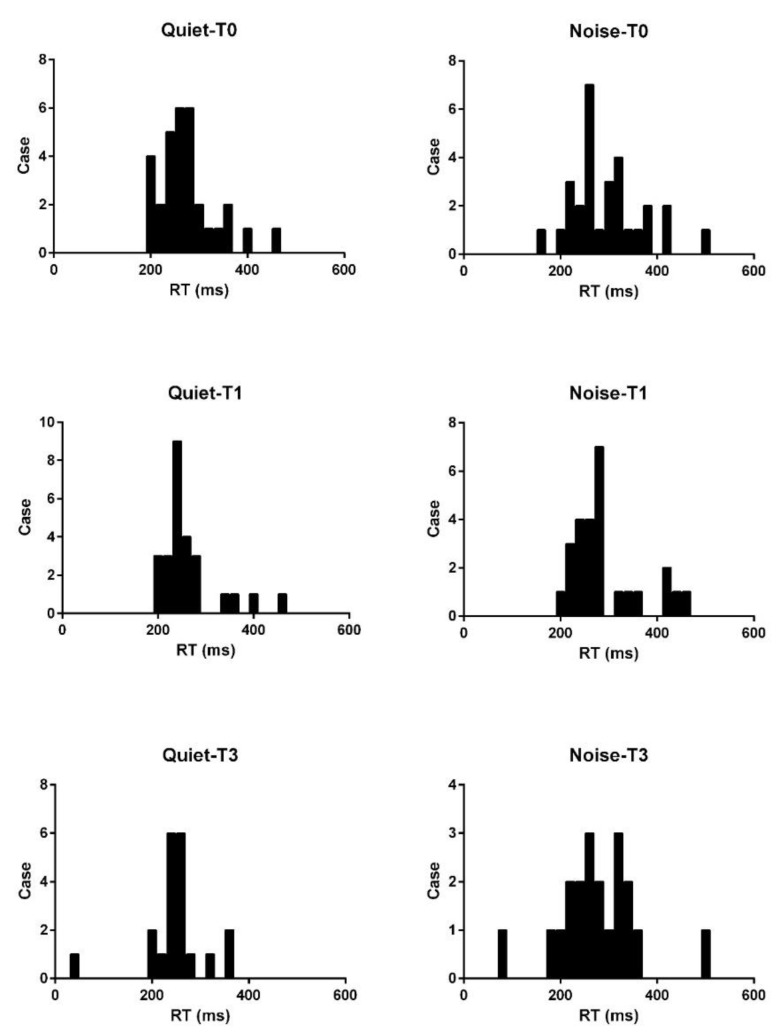
Histograms of reaction time (RT) distribution in quiet and noisy conditions before treatment (T0), 1 month after treatment (T1) and 3 months after treatment (T3).

## Appendix C

Preliminary data: healthy, tinnitus-free subjects
Subjects: 28
Male: 14; Female 14
Age (years): 38.50 ± 12.41; (range = 20–55)
Listening effort (visual reaction time): Quiet: 249.80 ± 48.90 ms; Noise: 257.27 ± 59.28 ms.

**Table A1:** Hearing results: the averaged SRT and PTA in each frequency of both sides.

(Hz)		SRT	250	500	1k	2k	4k	8k
Left	Average	13.39	14.11	13.04	12.68	10.89	14.82	15.18
	SD	5.94	6.95	5.50	6.73	8.17	9.86	15.96
Right	Average	13.21	15.71	14.29	13.21	11.96	15.18	16.25
	SD	5.97	6.63	6.77	6.12	7.50	9.86	11.99

SRT: speech recognition threshold; PTA: pure tone audiometry.

**Table A2:** Correlation analysis of Listening effort and SRT/PTA frequencies in left side.

Left Side (Hz)			SRT	250	500	1k	2k	4k	8k
Listening effort	Quiet	r	0.1038	0.2316	0.2590	0.2850	0.1821	0.2204	0.2028
		*p*	0.5991	0.2356	0.1832	0.1415	0.3537	0.2598	0.3007
	Noise	r	0.2068	0.2584	0.2300	0.3635	0.2507	0.2988	0.3388
		*p*	0.2911	0.1842	0.2391	0.0572	0.1981	0.1224	0.0778

r: Pearson correlation coefficent.

**Table A3:** Correlation analysis of Listening effort and SRT/PTA frequencies in right side.

Right Side (Hz)			SRT	250	500	1k	2k	4k	8k
Listening effort	Quiet	r	0.2029	0.09965	0.1134	0.1895	0.1517	−0.05045	0.1852
		*p*	0.3005	0.6139	0.5655	0.3340	0.4409	0.7987	0.3455
	Noise	r	0.2967	0.1586	0.05614	0.3251	0.2184	0.006638	0.2751
		*p*	0.1253	0.4201	0.7766	0.0914	0.2642	0.9733	0.1565

r: Pearson correlation coefficent.
